# Microbial-Based Products to Control Soil-Borne Pathogens: Methods to Improve Efficacy and to Assess Impacts on Microbiome

**DOI:** 10.3390/microorganisms11010224

**Published:** 2023-01-16

**Authors:** Magdalena Ptaszek, Loredana Canfora, Massimo Pugliese, Flavia Pinzari, Giovanna Gilardi, Paweł Trzciński, Eligio Malusà

**Affiliations:** 1 The National Institute of Horticultural Research, 96-100 Skierniewice, Poland; 2 Research Centre for Agriculture and Environment, Council for Agricultural Research and Economics, 00184 Roma, Italy; 3 Agroinnova, University of Torino, 10124 Torino, Italy; 4 Institute for Biological Systems, National Research Council of Italy, 00010 Montelibretti, Italy; 5 Life Sciences Department, Natural History Museum, London SW7 5BD, UK; 6 Research Centre for Viticulture and Enology, Council for Agricultural Research and Economics, 31015 Conegliano, Italy

**Keywords:** biocontrol, mycorrhiza, phenotypic arrays, analytical methods, formulation strategies

## Abstract

Microbial-based products (either as biopesticide or biofertilizers) have a long history of application, though their use is still limited, mainly due to a perceived low and inconsistent efficacy under field conditions. However, their efficacy has always been compared to chemical products, which have a completely different mechanism of action and production process, following the chemical paradigm of agricultural production. This paradigm has also been applied to regulatory processes, particularly for biopesticides, making the marketing of microbial-based formulations difficult. Increased knowledge about bioinocula behavior after application to the soil and their impact on soil microbiome should foster better exploitation of microbial-based products in a complex environment such as the soil. Moreover, the multifunctional capacity of microbial strains with regard to plant growth promotion and protection should also be considered in this respect. Therefore, the methods utilized for these studies are key to improving the knowledge and understanding of microbial-based product activity and improving their efficacy, which, from farmers’ point of view, is the parameter to assess the usefulness of a treatment. In this review, we are thus addressing aspects related to the production and formulation process, highlighting the methods that can be used to evaluate the functioning and impact of microbial-based products on soil microbiome, as tools supporting their use and marketing.

## 1. Introduction

The history of modern scientific research on the application of beneficial bacteria and fungi in agriculture began in the XIX century. The first studies on the use of microorganisms concentrated on plant growth stimulation and biological control [1,2,3], also fostering production and world-wide application of formulations [4,5,6]. Recent market analysis reports valued the global biopesticide market at about 10.2 billion USD by 2025 [7], and at 3.15 billion USD by the end of 2026 that of the biofertilizers market [8].

However, despite this history and the potential of microbial-based product application, their use is still limited, mainly due to a perceived low (or lower compared to chemical formulations) and inconsistent efficacy under field conditions [9,10,11]. Such perception could be ascribed to several factors, not all directly associated with the inoculated strain performance. Formulation processes, including the biotechnological method of production, the availability of bioinocula composed by a single strain or consortia, the application method, farmers’ and advisors’ knowledge in managing bioinocula, and regulatory and quality issues, are all factors that contribute to achieving an effective performance of microbial-based products for the control of soil-borne pathogens. In this review, we are addressing some of these aspects, highlighting the methods that can be used to evaluate the functioning and impact of bioinocula on soil microbiome, particularly on the mycobiome, which are considered tools that support their use.

## 2. Improving Production and Formulation of Bioinocula

**Figure 1:**
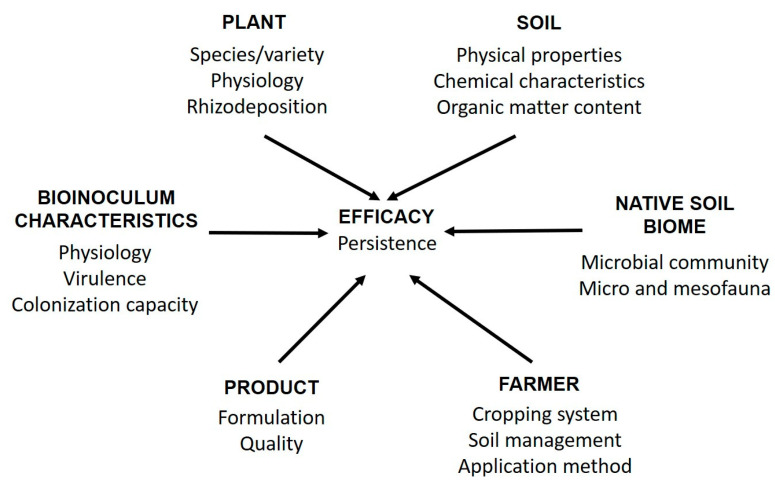
Factors affecting the efficacy of microbial-based formulations.

**Figure 2:**
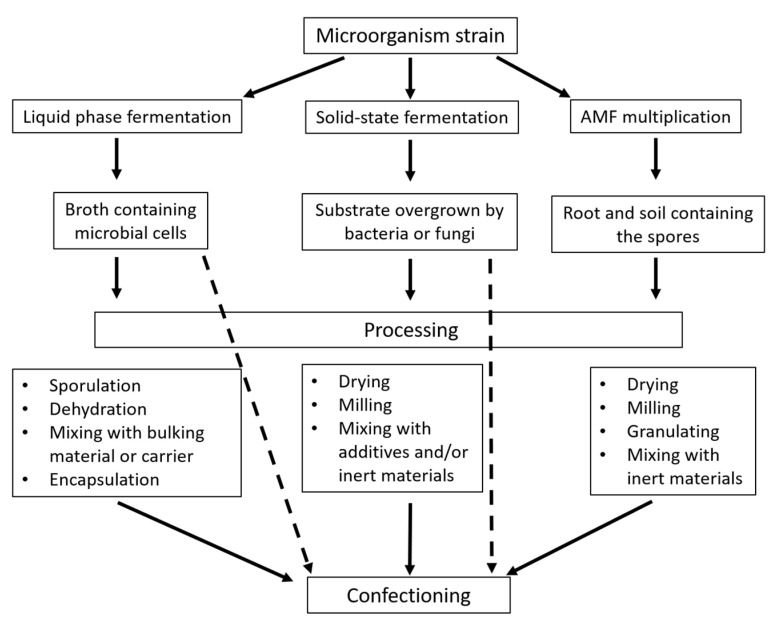
Biotechnological processes to produce microbial-based formulations. Dotted arrows show possibilities of direct packaging/use without the need for other processing steps.

The effectiveness of a microbial-based product for bioprotection or biofertilization depends on a combination of factors, including the properties of the microbial strain, its relationship with the specific crop plant, the production process, the formulation of the product, and the application method (Figure 1). Obtaining an economically viable and stable formulation with a high concentration of cells of the bioactive microbial strain can be challenging (Figure 2). Indeed, the physical and chemical parameters allow for reproducible results, and the selection of substrates for production shall consider their availability and cost [12,13]. Byproducts of the food industry (e.g., molasses or corn steep liquor) are commonly utilized to prepare liquid media while fruit and vegetable pomace can be used for solid-state fermentation [14,15,16].

Even though many strains have been isolated and proven to improve soil health and plant growth, only microorganisms capable of long-term storage are often selected to produce commercial bioinocula. This means that only a small part of bacteria and fungi with beneficial features can be used in practice: the majority of them being spore-producing bacteria, e.g., *Bacillus* spp., symbiotic bacteria, e.g., *Azotobacter* spp., or conidia-forming fungi, e.g., *Beauveria* spp., *Metharizium* spp. or *Trichoderma* spp. Few gram-negative, non-sporulating growth or health-promoting bacteria, e.g., *Pseudomonas* spp., are used in commercial products [17,18]. Mycorrhizal fungi represent a particular case, as their production can be obtained as crude inoculum from colonized roots of plants [19] or through a biotechnological approach exploiting root symbiosis [20]. In contrast to AMF, bacteria can be commonly cultivated in any multiplication process such as liquid or solid phase fermentation [14]. The biomass produced in liquid phase fermenters during cultivation can be and are easily included in formulation processes, e.g., condensation and inclusion in a carrier, dehydration (lyophilization or spray drying). Filamentous fungi and *Streptomyces* spp. are better suited to cultivation on solid media [14,15,16,17,18,19,20,21,22], which can deliver ready-to-use formulations [16]. A summary of the features and differences between solid-state fermentation and liquid-phase fermentation processes is presented in Table 1.

The price of microbial-based products has been frequently mentioned as a possible obstacle to their broad use in agricultural practice, prompting efforts to reduce production costs to foster the application of microbial formulations. Even though accurate calculations about the production costs of commercial formulations are lacking, media formulated with waste or by-products could contribute to reducing production costs of both solid-state and liquid fermentation processes [23,24,25]. Nevertheless, it is noteworthy that raw materials only constituted about 25% of the total production costs of a common bacteria for enzyme production, while the incidence of the facilities amounted to 45% [26], making it very difficult to estimate a general “average” cost for such kind of productions.

The storage of microbial-based products usually depends on their formulation: liquid products generally have a shorter shelf life than solid formulations and often require storage at lower temperatures [12]. Dehydration is one of the best options to extend the survival of microorganisms and makes the formulation less prone to contamination during storage time and more resilient to withstand environmental changes (e.g., temperature fluctuations). This method is the only one allowed to commercially produce microbial-based fertilizers under the new European Union legislation (which classifies them as “microbial biostimulants”). However, freeze-dried products should be stored in airtight packaging due to the possibility of absorbing water from the atmosphere [14,27]. Cell encapsulation with different carriers presents several positive features and advantages over other methods of formulations: it provides cell protection from adverse environmental conditions, also during storage and transport, it ensures high survival rates for up to a few years [28,29,30], can be used for any microorganism [31] and it is safe for the environment [32]. Formulation with different additives [28] and organic materials (e.g., compost—see Section below) can further improve the strain performance. Innovative formulations based on multilayer beds with the addition of protectants enhancing the protection of the bioinoculum from abiotic stresses have also shown promising results [33,34]. A possible disadvantage of formulations based on encapsulation in biopolymers derives from the release rate, which could be too low to impact plants or suppress the pathogen. Calculations made from trials with alginate beds showed that the release rate within 24 h would account for only 0.008 to 0.2% of the total bacterial population present in the formulation [35]. Over time, the overall population’s decrease in the formulation would also reduce its efficacy [36].

Field application of microbial-based products can represent a major limitation in their correct use as microbial plant growth promoters or for protection from soil-borne pathogens [10,37]. The type of device used for applying bioinocula will depend on the specific needs of the application and the characteristics of the formulation. There is no specialized equipment that would enable the liquid application of these products, thus sprayers for chemical pesticides are normally utilized for this scope. However, it was demonstrated that the prolonged working time of an ordinary common sprayer based on hydraulic atomization reduces the number of live cells by up to 50% [38]. The simplest method to apply both plant growth promoters and products to protect against soil-borne pathogens is on the roots during planting (e.g., by drenching), or using various devices, such as sprayers, injectors, or drip irrigation systems. Plant growth promoters and microbial pesticides can be coated directly on the seeds [39]. In the case of AMF formulations, due to the mechanism of infection of plant roots, the most effective method is to apply them near the root zone, for example during seedlings production or transplantation [40]. Some difficulties may arise for the application of bioinocula close to the root zone of multiannual crops (e.g., strawberry or fruit crops) due to the lack of equipment that allows applying the spores or crude inoculum into the ground in the vicinity of the root system. However, devices for application near the root system are currently under development and should overcome the drawbacks of standard machines for soil fertilizers or pesticide application [41].

Biocontrol with the use of microorganisms has generally consisted of using a single strain of bacteria or fungi [42,43], also as a result of regulatory approaches that have been considered microbial-based products similar to chemical compounds. The list of “active substances” based on microorganisms currently authorized at the European Union level (https://food.ec.europa.eu/plants/pesticides/eu-pesticides-database_en, accessed on 27 December 2022) counts 73 microbial strains that are individually registered and formulated (additionally, 24 are under evaluation). Examples of this approach include products based on *Trichoderma asperellum* (e.g., Xilon WP—Biocontrol Technologies, Barcelona, Spain), *Coniothyrium minitans* (Contans WG, Bayer Crop Science, Monheim am Rhein, Germany), *Bacillus thuringiensis* subsp. *kurstaki* (e.g., Lepinox Plus, CBC Europe, Varedo, Italy) or *Beauveria bassiana* (e.g., Naturalis, CBC Europe, Varedo, Italy). Only one product based on *Trichoderma asperellum* and *Trichoderma gamsii* has been registered instead (Remedier, Gowan, Yuma, AZ, USA). In the US, the same list includes 133 strains (https://www.epa.gov/ingredients-used-pesticide-products/biopesticide-active-ingredients, accessed on 27 December 2022). However, the current trend in plant protection research promotes the use of bioinoculants containing a mixture of microorganisms with different modes of action (e.g., mycoparasitism, competition, antibiosis, or induction of plant systemic resistance) or multifunctional (i.e., supporting plant growth and health). The presence of different bioactive species is thought to increase the application’s effectiveness and extend the spectrum of the product’s efficacy toward various pathogen species [44,45,46,47]. However, the design of a microbial consortium is challenged by the interaction and mode of action of the potential strains [48,49,50,51]. The consortia could be designed as a mixture of different strains belonging to the same species or composed of species of different genera [45,46]. However, this scenario is absent from any legal provision dealing with the marketing of bioproducts; instead, in the European Union, the criteria for the classification of a bioinoculum as pesticide or plant biostimulant discriminate the two purposes, making it unrealistic to believe that multifunctional products will be marketed in the near future [52]. Moreover, the new EU legal framework for fertilising products has included among the microbial-based products (classified as microbial biostimulants) only four groups of microorganisms, namely *Rhizobium* spp., *Azotobacter* spp., *Azospirillum* spp., and mycorrhizal fungi, thus not allowing any claim other than that for biostimulation related to plant nutrient uptake [53].

## 3. Methods for the Study of Bioinocula Interactions with Soil Microbiome to Increase Their Efficacy and Marketing

A significant challenge in the formulation process of microorganisms’ consortia is to untangle the interactions among the strains that compose the consortium. Evaluating the species interaction in a consortium and deciphering the dynamics within the establishment of coexistence in microbial-based product design is a complex task [54]. Although microorganisms live in nature within complex ecological communities [55,56,57], assembling a synthetic microbial consortium [58] within a growth substrate can provoke unexpected antagonistic or not beneficial behaviors that compromise the bio-production process [59]. Therefore, bioinocula species interaction shall be accounted for in the formulation process, as the nature of these interactions can vary depending on substrate components utilization and nutrients’ needs: a better understanding of the triggering or depressing-response mechanisms can improve the formulation efficacy.

**Figure 3:**
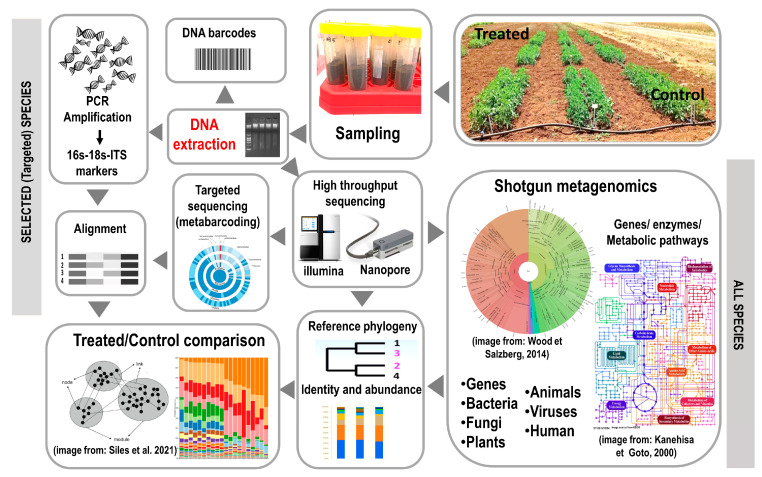
The diagram shows the techniques based on DNA that can be used to evaluate the impact of bioinoculants on soil microbial communities. Soil samples must be obtained from comparable field trials, where most environmental variables could be controlled. DNA should be extracted from replicated samples (biological replicates) possibly made of grams of soil (not micrograms). Extracted DNA can then be used to amplify diagnostic sequences by PCR, and microorganisms can be identified by matching their diagnostic sequences (barcodes) with sequences deposited in public databases. Once amplified, DNA can be analyzed by massive sequencing. High-throughput sequencers also allow the use of protocols in which DNA is not first amplified, and all the extracted material is sequenced. In this case, we speak of metagenomics, a technique that allows obtaining information on genes and enzymes possessed by microorganisms but also viruses and macroorganisms that in any form entered the soil, releasing their DNA [63,64,65].

The complexity of interactions between microorganisms of a bioinoculum consortium requires a step-by-step approach in their study, which cannot ignore the taxonomy and the characterization of the species, including the cell size and the behavior of the single cell in a specific growth medium [60,61], as well as their phenotype, which is characterized by the association to a defined host and the type of interaction (beneficial, antagonistic) [62]. Several methods can be applied to study microbial species interactions [59], to obtain more detailed profiles and achieve more significant insights into species interactions within a consortium (Figure 3).

Microplates (e.g., Phenotype MicroArray) can be used to compare the metabolism of multiple species in terms of potential niche overlap [66,67], i.e., to test individual strains in terms of carbon and nitrogen sources utilization [68,69,70]. Applying statistical or mathematical models to the data on metabolic dynamics, it is possible to predict the likelihood of two species competing once they are grown together. Another way of using phenotype microplates is by inoculating together two or more strains forming a potential consortium. The result allows the verification of the interaction between species, and the design of nutrient substrates in which the species do not compete, or at least produce abundant biomass despite coexistence and often competition. Phenotype microarray techniques have been applied under a wide variety of nutrient and growth conditions, including NaCl tolerance and antibiotic resistance, to study bacteria (both gram-positive and gram-negative), yeasts and filamentous fungi to be employed as bioinocula in different fields of research [62,71,72]. Pinzari et al. [73] analyzed and discussed the potential of Biolog^®^ Phenothype Microarraysto investigate functional diversity, niche overlap, and catabolic versatility of several fungal co-inoculates compared to single inoculum, concluding that this is a valuable approach to obtain insights into species interaction.

However, when more than one species of fungi or bacteria are inoculated together, the problem arises of quantifying their relative abundances and defining which microbial species introduced into the system contributed to the observed effect and in what proportion the different species benefited, e.g., by developing biomass. Hence, in the case of co-inoculation, it becomes indispensable and more meaningful to combine phenotypic plates with a species-specific quantification system, i.e., DNA-based methods [74], particularly the qPCR analysis. This analysis allows quantifying the share of each species in the co-inoculated microplates, making it possible to predict the type of interaction between the co-inoculants in different nutritive conditions. For example, the combination of Phenotype MicroArray with the use of SSR markers and Real-Time qPCR analysis allowed the evaluation of the behavior of the co-inoculum of two entomopathogenic fungi compared to their single inoculum [75], showing the suitability of the methodological approach to assess the performance and potential competition of co-inoculated beneficial strains. Species/strain-specific marker/s capable of discriminating between two or more microbial species are needed to obtain a reasonable quantification of the bioinoculants from the Phenotype MicroArray wells. Nevertheless, even if accurately quantified, it is not possible with DNA-based methods to distinguish between alive and dead cells. This is particularly important also when monitoring the bioinocula in complex matrices, such as soil, or, in general, when estimating the relationship between the overall diversity of a microbial community and its active (i.e., living) fraction alone [76].

This bottleneck can be overcome utilizing methods based on the extraction and quantitation of ribonucleic acids (RNA), which are powerful techniques to obtain insight into functional trait expression in living cells and active organisms, as RNA is rapidly degraded upon cell death [77]. However, RNA-based methods are expensive and time-consuming. Numerous studies have relied on propidium monoazide (PMA), which binds the DNA of cells no longer alive, to discriminate between dead and viable cells. Studies combining qPCR with PMA dying have been conducted to evaluate the relative abundance of target microbial genes within the living population of cells in an array of tissues and matrices [78,79,80,81].

The interaction between microorganisms assembled in a co-inoculum can be studied with plate cultures or in multi arrays [82]. However, if the bioinoculum is used in soil, it may be helpful to consider interactions over short distances and in more physically complex situations than an agar plate [82]. Among the effective and novel techniques for studying interactions between fungi or between fungi and bacteria are microfluidic systems. These miniaturized systems have been successfully applied in specific domains of microbiology [83]. Microfluidic chips make it possible to simulate soil structure and microhabitats or to directly observe soil micropores and the dynamics of microorganisms in compartmentalized and controlled systems [84,85]. These devices have been used to demonstrate the movement of bacteria along fungal hyphae [86], to document the interaction between fungi and parasitic nematodes, or the mechanisms of competition between antagonistic fungi [61]. The application of microfluidics to mycology is a more recent development [87,88]. The use of microfluidic systems for studying fungi, also called ‘Fungi-on-a-Chip’, involves manipulating small amounts of liquid in a controlled manner within micron-sized artificial fluidic networks. In general, miniaturization makes it possible to generate high-performance experimental systems with greater analytical accuracy and sensitivity, allowing to control processes, especially in terms of temperature, illumination, or flow dynamics. Microfluidic chips offer the optical transparency of most bright-field and fluorescence imaging devices and the ability to mimic microenvironments structurally and with well-defined chemical gradients. The choice of material is crucial for the desired application, and the elastomeric polymer, poly(dimethylsiloxane) (PDMS), is one of the most widely used materials in developing microfluidic technology. The miniaturized system can be built using plastic supports suitable for observation under optical and electron microscopy (i.e., poly(dimethylsiloxane)) [88].

Recently, Gimeno et al. [61] developed a system based on a microfluidic channel device coupled with scanning electron microscopy image analysis allowing the quantification of hyphal growth and monitoring the localized and systemic effects of bioinocula, relevant for the development of bioproducts. They also combined the image analysis with an enzymatic assay to better investigate the fungal interactions, opening a new methodological approach for qualitative and quantitative analysis of microbial species interaction.

## 4. Methods for Evaluating the Effect on the Soil Microbiome to Improve Microbial-Based Product Exploitation and Environmental Impact Assessment

An effective bioinoculant must impact the species already present in the soil, particularly the plant pathogens [89]. Therefore, introducing bioinoculants into an agroecosystem creates the need to assess their impact on the soil native microbiome and estimate their efficacy against the populations of plant pathogens. The answer to such questions also averts undesirable effects, such as an excessive impoverishment of natural biodiversity or competition with fungal or bacterial species whose presence is desirable for crops, such as certain mycorrhizal fungi or nitrogen-fixing bacteria [89,90]. Nevertheless, the physiological characteristics of the bioinoculum determine to a great extent its survival/fate and activity in the soil and biotic/abiotic soil factors are also major factors affecting the persistence/decline of a microbial inoculant population introduced in the soil as well as its contribution to the provision of ecosystem services [91].

Analyzing the impact on a complex ecosystem, such as the soil, involves using techniques different from those applied to study the in vitro interaction between the introduced microorganism(s) and the target plant pathogen. In fact, different species with similar functions may coexist in the soil that, despite a close genetic base, show variability in the type of response to the inoculation [92]. The analysis of a bioinoculant’s effect on the soil’s biodiversity and functionality can be approached with different levels of complexity (and cost).

A bioinoculant’s qualitative and quantitative impact on an agroecosystem’s microbiome can be assessed by studying total soil DNA [93]. Today’s most cost-effective techniques for such evaluation are based on the amplification of target sequences from the extracted DNA (marker genes such as 16S for bacteria or ITS for fungi) using the polymerase chain reaction (PCR) [94]. The 16S rRNA gene consists of highly conserved nucleotide sequences interspersed with some variable regions that are genus- or species-specific [95]. In the case of fungi, the ITS (Internal Transcribed Spacer) of nuclear DNA has become the most sequenced region to identify fungal taxonomy at the genus level and eventually within species [96]. Comparing the individual DNA sequences of bacteria and fungi with those stored in public databases makes it possible to construct phylogenetic relationships between microorganisms and identify them by similarity to sequences of already identified species [95,96].

In the last decade, several new methods for DNA sequencing have been developed named ‘next-generation’ or ‘second-generation’ sequencing (NGS) platforms to distinguish them from earlier systems such as Sanger sequencing [97]. These technologies have enabled the implementation of High Throughput Sequencing (HTS) molecular techniques [98]. There are roughly two main HTS techniques applied to study the impact of bioinoculants on agricultural ecosystems: targeted sequencing and metagenomic shotgun sequencing [99]. The targeted sequencing method involves the amplification and subsequent sequencing of a target gene sequence. A DNA sequence that provides taxonomic information and is common to all organisms of interest is used as a ‘barcode’ or genetic marker and amplified by PCR [100]. The amplicons obtained are then massively sequenced and bioinformatically characterized to determine which microorganisms are present in the soil sample and their abundance. This technique yields qualitative data useful to identify species in the sample and quantitative data on their abundance. However, targeted sequencing is limited to the analysis of taxa based on genetic markers available from databases [100]. Suppose a microbial species has never been isolated or identified, no marker sequences would have been deposited in a public database, making it impossible to identify the organism by a metabarcoding analysis. The analysis would thus result in a number of sequences (OTU) corresponding to an unknown species with unknown properties.

The second and more powerful HTS technique obtains sequences of the total DNA (the metagenome) extracted from a sample, not just that of selected microorganisms’ genetic markers. The study of metagenomes (called ‘metagenomics’) [101,102], is an emerging field in microbial ecology, as the power of the analysis of the entire DNA of an organism is applied to the whole community of microorganisms, overcoming the need to isolate and cultivate individual microbial species. In its approach, metagenomics transcends the individual genome, providing an enormous amount of data which allows quantifying the microbial community diversity in terms of species richness/abundance [101,102]. The use of highly sensitive alignment algorithms to elaborate the sequence data makes it possible to identify many genomic sequences showing similarities to those already studied, thus allowing to assess the abundance of each organism in any soil sample and the genes coding for enzymes and proteins of the same organism.

In the shotgun approach with targeted sequencing, all DNA is fragmented and sequenced independently, and no PCR is performed. The results obtained with the shotgun method also include the type of information obtained with the targeted method [103]. The sequences obtained (reads) with the shotgun approach are quality controlled and aligned to various genomic sequences in public databases. With this method, it is possible to analyze the microorganisms present in the sample, their abundance, and the type and abundance of genes coding for specific proteins. It is also possible to reconstruct the metabolic pathways potentially expressed in the sample by determining the set of genes coding for a specific process [104].

Recently, third-generation sequencing systems have become available on the market, which provide longer and more informative DNA sequences than ‘second-generation’ sequencers and are increasingly being adopted by small and non-specialized laboratories. In particular, Oxford Nanopore Technologies (ONT) provides a miniaturized sequencer (MinION) supported by easy-to-use kits and online bespoken bioinformatics platforms that are used for targeted sequencing and shotgun protocols and have been successfully applied to the study of microbial communities of soils subjected to different treatments [105].

Bioinformatic analysis of data derived from metabarcoding and metagenomics studies can be performed in several ways [103]. Many protocols, both public and associated with paid software, are currently available [106]. However, all protocols must, at a certain point, make use of curated public databases to obtain taxonomic and functional profiles [107]. Public phylogenetic and functional databases often lack data about species of interest for soil biodiversity studies, which are also poorly represented in collections of living cultures [108]. Efforts to populate databases, such as GenBank (https://www.ncbi.nlm.nih.gov/genbank/, accessed on 27 December 2022), with culture-based verified diagnostic sequences obtained from rhizosphere microorganisms of major crops could improve future amplicon-based metagenomics studies related to the application of bioinocula. Indeed, without bacterial and fungal annotated genomes, it is challenging or impossible to match taxonomic identification with DNA sequences stored in databases.

The annotation of the sequences of a genome is a multi-step process that maps a gene function to the genome. It begins by aligning sequences by similarity with genomes of related species already annotated to protein-coding genes and then to other functional units of the genome (e.g., structural RNAs, tRNAs, small RNAs, pseudogenes, control regions, direct and inverted repeats, insertion sequences, transposons, and other mobile elements). In the case of reference databases for metagenomic analyses, the genome of a reference microorganism allows annotating reads corresponding to protein sequences [109]. Nevertheless, also in this case, the annotated genomes of fungal and bacterial soil species available in public databases are only a few, which represents a significant limitation in applying shotgun metagenomic analysis to bioinoculant impact evaluation. Such a drawback could be overcome by regulatory requirements that ask for microorganisms to be marketed as microbial-based products to have their genome sequenced, annotated, and uploaded to public databases.

In addition to the paucity of data from species of interest for soil bioinocula in the databases, there is also a scarcity of bioinformatics tools that can combine genetic and biochemical data or that can support the modelling of interaction mechanisms between soil species or between functional groups, which would favor predicting their impact or effects on the soil microbiome [110]. Network analysis [63,111] and other methods based on artificial intelligence algorithms capable of comparing complex patterns could be effectively applied to screen the impact of bioinoculants on soil communities.

Both metabarcoding analysis and metagenomic analysis applied to study the impact of a bioinoculum on soil microbiome must take into account certain known limitations of these techniques [112,113,114]. In particular, the significant variability of the structure and organization of the soil microbiome, depends on the scale of observation, soil physico-chemical characteristics, and seasonal patterns [115,116]. Therefore, some aspects should be carefully considered when using molecular techniques to compare soil before and after applying a microbial bioinoculum [117]. In particular, it is advisable to analyze many replicates to cope with the spatial variability typical of soil; to extract DNA from large soil samples (i.e., 10 grams of soil) to have greater representativeness of the species contained in the analyzed volume, to repeat the analysis at intervals of days or months, and to take into account the climatic conditions at the time of sampling [118,119].

Even though HTS methods can be powerful in capturing species diversity and abundance, their application in the study of the impact of bioinoculants on soil ecology and functionality is never straightforward and rarely fully effective. Once counts of genes (in the case of metagenomics) or taxa (in the case of both metagenomics and metabarcoding) have been obtained, it is necessary to compare different microbial communities with each other (e.g., before and after the bioinoculant application or at different times after the application). There are various approaches for comparing communities, both on an overall level and for specific genes and taxa [120]. These are generally statistical and bioinformatic techniques, using, for example, dissimilarity measures between each pair of samples (i.e., communities), which can be compiled into a distance matrix. Statistical techniques range from Euclidean distance calculation to more sophisticated approaches such as Bray-Curtis dissimilarity [121] or the Jaccard index (quoted in Bengtsson-Palme, [120]). The resulting distance matrices can be used as input for statistical methods such as Mantel’s test [122] or Anosim [123] or Permanova [124]. Data exploration methods such as principal coordinate analysis (PCoA), non-metric multidimensional scaling (NMDS), and various clustering systems helpful in identifying groups of genes and taxa that co-occur under the conditions determined by the experimental conditions are commonly used. These approaches applicable to both functional and taxonomic data are largely implemented in the Vegan R package for ecological analysis [125]. There are also approaches to compare large datasets developed initially for differential gene expression analysis, such as those represented by the bioinformatics packages edgeR [126] and DESeq [127] that use non-parametric tests [128], which are less sensitive to the variability of metagenomics datasets and thus more robust to outliers. Co-occurrence network analysis techniques can be helpful, for example, in assessing the number of interactions between taxa or between the introduced organisms and the species already present in the environment [129].

However, even when obtaining statistically significant information on the effect of a bioinoculum on other soil taxa, the interpretation of such results is not always straightforward [130]. If adding the bioinoculum corresponds to a decrease in the diversity and abundance of taxa considered pathogenic to the crop, it would be possible to attribute a positive effect to the interaction [131]. However, although clear from the data, the effects are very often difficult to interpret, partly because little is known about the role of most of the taxa in the community. The search for nutritional or physiological factors correlating with taxonomic or functional data is sometimes an effective strategy that allows drawing direct relationships between the bioavailability of certain nutrients and the presence/size of specific taxa or defined functional gene clusters [132].

## 5. Probiotics and Prebiotics to Control Soil-Borne Pathogens

Key strategies for the microbial management of soil-plant systems and consequently the control of soil-borne pathogens can be based on the use of biostimulants (i.e., prebiotics), which are products able to improve microbial diversity and soil microbial health by promoting the growth of soil microorganisms already present within the soil-plant system, and on the application of beneficial microorganisms (i.e., probiotics), which exert health promoting and nutrient-mobilizing properties [133].

The impact of an individual or a consortium of bioinoculants can be measured if any modification occurs in the microbial communities as they interact with the plant rhizosphere, where they can enhance the soil nutrient availability or uptake and biotic stress tolerance of plants, either through induced soil suppressiveness or by inducing systemic tolerance [134]. The soil microbiota can be manipulated with bioinoculants to reduce either pathogen inoculum or its virulence in conducive soil, although the effectiveness of these approaches depends on the specific pathogen/host system [135,136].

Moreover, certain abiotic factors (e.g., pH and soil type) play important roles in driving the microbiota dynamics and soil suppressiveness against soil-borne pathogens of vegetable crops [136]. In field trials against lettuce fusarium wilt in naturally or artificially infested soils, Bellini et al. [137] showed that the microbiota composition at genus/class levels of the rhizosphere was affected more by the soil type than by the experimental treatments done with *Trichoderma* spp. and *Bacillus amyloliquefaciens*. When considering the tomato-*Fusarium oxysporum* f. sp. *lycopersici* pathosystem, it was observed that microbial inoculants introduced into the soil as strains or through compost treatments, enhanced the populations of beneficial microorganisms (e.g., *Bacillus* spp. and *Trichoderma* spp.) and fostered a marked negative correlation with fusarium wilt severity [138]. Similar results were found for zucchini-*Phytophthora capsici* and lettuce-*F. oxysporum* f. sp. *lactucae* pathosystems [138,139].

However, the impact of microbial inoculants depends also on the application method and/or formulation used (Table 1). In this respect, different strategies could be planned to reduce the damage from soil-borne pathogens using probiotics, prebiotics, or synbiotics [133]. Bioinocula can be considered probiotics for the soil-plant system, exerting health-promoting and nutrient-mobilizing properties on plants [140]. Prebiotics (e.g., compost, humus, animal manure, etc.) improve microbial diversity and soil microbial health by promoting the growth and diversity of native soil microbial populations within the soil-plant system [141,142]. Composts can be considered also a synbiotic products [143] as microbial strains could be additionally inoculated into them.

The enrichment of organic fertilizers and soil improvers (e.g., compost) with microbial inoculants were a good strategy to manage *P. capsici* on zucchini, as it improved the quality of the rhizosphere microbiome [144]. Moreover, applying a compost enriched with bioinoculants against the *Fusarium* wilt of lettuce was found to be more effective than applying the bioinoculants on their own [145]. Seed treatment is also considered a suitable method for introducing microbial-based products into the soil to control specific pathogens, as it allows colonizing the rhizosphere from the initial phases of root development and promotes plant-microorganisms relations by exploiting the root exudates [50,146,147,148]. Microbial-based products introduced into the rhizosphere of planting material as a preventative treatment in a nursery or applied at transplanting should also support the development of stable microbial populations in the seedling rhizosphere, also favoring their establishment in the field. Soil inoculation with microorganisms in the presence/absence of indigenous soil-borne pathogens induces host-specific changes in the plant and related soil microbiome, causing short-term shifts to improve or repair a healthy plant microbial community in the long term [149,150,151].

Microbial consortia appear to be more effective than individual microbial isolates with different vegetable crops [152] (Table 1), even though in a few cases they were less effective or were as efficient as individual strains [153,154,155,156]. For instance, the introduction of functionally diverse consortia of *Pseudomonas* improved their establishment, survival, and ability to control *Ralstonia solanacearum* into the natural rhizosphere microbiome of tomato than a single strain or species, also because a greater variety of toxins were produced [157]. A consortium composed of *P. aeruginosa*, *T. harzianum*, and *B. subitilis* resulted in suppressing soft-rot pathogen *Sclerotinia sclerotiorum* compared to untreated control more than each individual strain [158]. A consortium composed of yeast (*Pichia guilermondii*) and a bacterium (*Bacillus mycoides*) significantly inhibited the occurrence of gray mold on strawberry leaves under different temperature conditions compared to the individual application [159]. Volatile organic compounds and tomato root exudates have also been involved in the control of *R. solanacearum* by *Bacillus amyloliquefaciens* GB03 through a plant-mediated microbiome shift [160]. The concept of community assembly of inoculants can be transferred to other fields of microbiome research and biotechnology [161], and has been proposed as a solution to improve industrial formulation processes for preparing synthetic microbial communities [162].

Arbuscular mycorrhizal fungi (AMF) are receiving growing interest as species promoting plant tolerance against several soil-borne pathogens [163,164] and plant-parasitic nematodes [165]. Co-inoculation of plant growth-promoting bacteria and AMF combines the benefits of each microbial component to increase crop productivity and disease control (Table 2). However, the degree of suppression varies between soils and involves both abiotic and biotic components [153,165,166]. Nevertheless, commercial AMF-based products do not have the possibility to be marketed in the European Union claiming “protection” effects, as they have to be registered as microbial biostimulants, a category of fertilizer products.

In addition to analyzing the impact of bioinocula on the targeted pathogen, attention should also be turned to the effect on non-target organisms, including higher organisms, as effects on non-target organisms depend on the mode of action of microbial inoculants [167]. This is specifically required by regulators, e.g., for registration in the EU [168], and companies have to provide data on non-target organisms and humans via the environment (concerning the exposure to the microorganism and to possible metabolites of concern produced by the strain). A review of the potential side effects on non-target organisms, such as predators, parasitoids, pollinators, and arthropods, posed by entomopathogenic fungi has shown no serious impact on them [169,170]. However, changes in soil microbial communities by bioinocula application may indirectly affect some insects that establish relationships with fungal species: for example, leaf-cutting ants that supply soil fungi with food and gain sustenance from their hyphae [171]. Considering these potential direct or indirect impacts of bioinocula and the regulatory requirements, the adequate monitoring of bioinocula using newly developed methods based on DNA is needed to better evaluate the overall impact on the environment and the ecosystem.

**Table 2:** Examples of soil-borne pathogens of solanaceous, lettuce and cucurbit plants that can be controlled using microbial-based products and biostimulants.

Crop/Pathogen System	Microbial Inoculant	Method of Application	Observed Effects	Impact on the Soil Native Microorganisms	Reference
Lettuce/*Fusarium oxysporum* f. sp. *Lactucae*	Green compost enriched with *Trichoderma virens* TW2. Combination of *Trichoderma gamsii* + *T. asperellum* with *Bacillus amyloliquefaciens* and potassium phosphite. Combination of *Trichoderma polysporum* + *T. atroviride.*	Preventative application in nursery and at planting in naturally or artificially infested soil.	Fusarium wilt severity reduction, in all cases and over the years, from 50% to 70%, compared to the untreated control.	The microbiome was not affected by the treatments, and no significant differences were observed when comparing the soil microbial community with that of the untreated control.	[137]
	*Bacillus subtilis* QST713 (Serenade Max). *Trichoderma gamsii* ICC 012 + *T. Asperellum* ICC 080 (Remedier). *Trichoderma virens* TW2. Mixture of three *Pseudomonas putida* strains (FC7B + FC8B + FC9B). Green compost enriched with *Trichoderma virens* TW2.	Preventative application in nursery and at planting in naturally or artificially infested soil.	Fusarium wilt reduction by as much as 69%.	Relevant impact of the treatments on ammonia-oxidizing bacteria and on the archaea that harbor the amoA gene. Significant negative correlations between *Bacillus subtilis, Trichoderma*, and *Pseudomonas* sp. abundances and wilt severity. No negative impact on the indigenous microbial communities.	[139]
Zucchini/*Phytophthora capsici*	Green compost enriched with *Trichoderma virens* TW2.	Mixed with the potting soil at different concentrations (1–10–20% *v*/*v*) in controlled greenhouse pot trials.	*Trichoderma*-enriched compost administered at 10% *v*/*v* reduced *P. capsici* by 50%, but when applied at 20% did not significantly suppress the pathogen.	Differences in population composition at genera level and in relative abundance according to the mycobiota sequencing. PCA analysis clustered the treated soils separately from the untreated ones.	[144]
Zucchini/*Phytophthora capsici*	*Bacillus subtilis* QST713 (Serenade Max). *Trichoderma gamsii* ICC 012 + *T. asperellum* ICC 080 (Remedier). *Trichoderma virens* TW2. Mixture of 2 *Trichoderma* sp. (FC7 and FC8). Green compost enriched with *Trichoderma virens* TW2.	Three soil applications with BCAs to the plug trays between sowing and transplanting. The microbial-enriched compost was mixed at 20% *v*/*v* in the tray and immediately before sowing.	All the treatments reduced disease severity by as much as 50%.	Alphaproteobacteria enrichment and, in particular, a more relative abundance of Bradyrhizobium, Mesorhizobium and Hypomicrobium, suggesting their involvement in disease suppression. No modification of the mycobiota, but. all the treatments reduced pathogen abundance.	[138]
Tomato/*F. oxysporum* f. sp. *lycopersici*	*Bacillus subtilis* QST713 (Serenade Max). *Trichoderma virens* TW2. Green compost enriched with *Trichoderma virens* TW2.	Four soil treatments with bioinoculants to plug tray between sowing and transplanting in a commercial nursery. Microbial enriched compost was applied twice: first mixed with the substrate at sowing, and then mixed with the soil one week before planting	The treatments reduced Fusarium wilt severity by as much as 50%.	No negative effects of the bioinoculants were observed on non-target microbial communities. Decreased *F. lycopersici* abundance in the soil with a greater abundance of the inoculated microbials and an accumulation of transcripts encoding PR genes.	[138]
Tomato/*Fusarium oxysporum* f. sp. *lycopersici*	*Trichoderma harzianum* *P. fluorescens* *Trichoderma harzianum* + *P. fluorescens* *Glomus intraradices* (AMF)	Seed coating.	Higher germination rate (22–48%) and lower mean germination time (less than 2.5 days) of tomato seeds than the control; the combination of bioinoculants were more effective than single-isolate treatments. The combined *P. fluorescence + T. harzianum,* or AMF provided a disease reduction of 67% in the field, compared to the non-treated plants.	The impacts were not evaluated.	[147]
Tomato/*Ralstonia solanacearum*	Fluorescent pseudomonad strains (CHA0, PF5, Q2-87, Q8R1-96, 1M1-96, MVP1-4, F113, and Phl1C2) were combined in different consortium to simulate different levels of strain richness.	Introduced multispecies probiotic consortia of Pseudomonas into a naturally diverse tomato rhizosphere microbiome, 5, 15, 25, and 35 days after the pathogen inoculation in greenhouse experiments.	The survival of introduced Pseudomonas consortia increased with increased diversity. The highest Pseudomonas diversity reduced pathogen density in the rhizosphere and decreased the bacterial wilt incidence.	The most diverse probiotic Pseudomonas communities composed of 8 strains were able to persist at high densities and to compete for resources with the pathogen and the natural bacterial communities.	[157]
Tomato and pepper/*Verticillium dahliae*	AMF combined with humic acids and/or whey	Pre-inoculation in growth chamber	Reduced wilt disease severity and *Verticillium dahliae* microsclerotia number.	The impacts were not evaluated.	[172]
Tomato/*Fusarium oxysporum* f. sp. *lycopersici*	*Funneliformis mosseae* *Glomus fasciculatum* *Bacillus velezensis* strain ERBS51 *Bacillus* sp. strain ERBS10	Applied alone or combined under pot and field conditions.	The combined bioinoculant (*F. mosseae* + *G. fasciculatum* + *B. velezensis* + *Bacillus* sp.) was the most effective in reducing the Fusarium wilt severity (−77.44%) followed by *F. mosseae* + *B. velezensis* + *Bacillus* sp. or *B. velezensis* + *Bacillus* sp. (−66.67%)	The impacts were not evaluated.	[173]

## 6. Conclusions

Production and application of bioinocula to improve plant nutrition and health is a highly promising field of research supporting the agricultural shift toward an economic and socially sustainable production with lower environmental impact. Co-cultivation and/or co-formulation of strains with different functions shall become the core of the overall production technology.

Following the human gut example, new strategies for the exploitation of beneficial microorganisms can be based on prebiotic, probiotic, synbiotic, and postbiotic products. A previous analysis of soil physical/chemical characteristics and microbiome dynamics along the plant growth, also as a function of the climatic-specific conditions, shall be a part of the overall assessment to determine the most efficient approach to take advantage of microbial-based products.

The development of multi-omics tools and interdisciplinary approaches to the derived data, eventually with the support of artificial intelligence, shall foster better exploitation of the native biodiversity and inoculated strains [174]. However, the microbial activity of soil, related to the introduction of microbial inoculants in the field through sustainable practices, should be monitored to evaluate the long-term impact of such inoculants against soil-borne pathogens. Guidelines for selecting species or strains that work together in performing a desired community-level function against soil-borne pathogens present in the host-associated microbiome are needed. Even though the knowledge in the field of microbial-based product application has been growing in the last few decades, there are still unexplored opportunities for therapeutic treatments of soil-borne pathogens implementing “holo-biotic” measures that could optimize the soil microbiome functioning for healthy crops.

## Figures and Tables

**Table 1:** Advantages and disadvantages of solid-state fermentation in comparison to liquid phase fermentation processes.

Advantages	Disadvantages
Higher productivity Better oxygen circulation Low-cost media Less effort in downstream processing Reduced energy and cost requirements Simple technology Scarce operational problems Resemblance to natural growing conditions for several microorganisms Broad range of applications (biocontrol agents, biofertilizers, composting) Less waste water	Lower effective mixing Difficult control of process parameters (pH, heat, moisture, nutrient conditions) Problems with heat build-up Higher impurity product, increasing recovery product costs Difficulties on scale-up Difficulties in the development of simple and automated bioreactors
